# Diversity Matters: Optimal Collision Energies for Tandem Mass Spectrometric Analysis of a Large Set of N-Glycopeptides

**DOI:** 10.1021/acs.jproteome.2c00519

**Published:** 2022-10-06

**Authors:** Helga Hevér, Kinga Nagy, Andrea Xue, Simon Sugár, Kinga Komka, Károly Vékey, László Drahos, Ágnes Révész

**Affiliations:** † MS Proteomics Research Group, Eötvös Loránd Research Network, Research Centre for Natural Sciences, Magyar Tudósok körútja 2, Budapest H-1117, Hungary; ‡ Chemical Works of Gedeon Richter Plc, Gyömríi út 19-21, Budapest 1103, Hungary; § Hevesy György PhD School of Chemistry, Faculty of Science, Institute of Chemistry, Eötvös Loránd University, Pázmány Péter sétány 1/A, Budapest H-1117, Hungary; ∥ Department of Chemical and Environmental Process Engineering, Budapest University of Technology and Economics, Budapest H-1111, Hungary

**Keywords:** tandem mass spectrometry, bottom-up proteomics, N-glycosylation, glycopeptide fragmentation, identification score, search engine, collision energy optimization, transferability

## Abstract

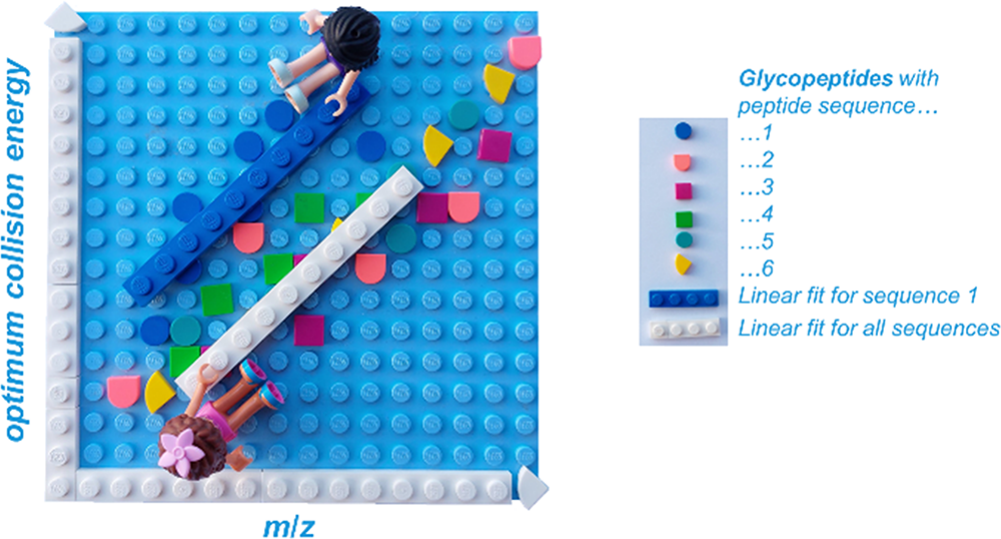

Identification and characterization of N-glycopeptides from complex samples are usually based on tandem mass spectrometric measurements. Experimental settings, especially the collision energy selection method, fundamentally influence the obtained fragmentation pattern and hence the confidence of the database search results (“score”). Using standards of naturally occurring glycoproteins, we mapped the Byonic and pGlyco search engine scores of almost 200 individual N-glycopeptides as a function of collision energy settings on a quadrupole time of flight instrument. The resulting unprecedented amount of peptide-level information on such a large and diverse set of N-glycopeptides revealed that the peptide sequence heavily influences the energy for the highest score on top of an expected general linear trend with *m*/*z*. Search engine dependence may also be noteworthy. Based on the trends, we designed an experimental method and tested it on HeLa, blood plasma, and monoclonal antibody samples. As compared to the literature, these notably lower collision energies in our workflow led to 10–50% more identified N-glycopeptides, with higher scores. We recommend a simple approach based on a small set of reference N-glycopeptides easily accessible from glycoprotein standards to ease the precise determination of optimal methods on other instruments. Data sets can be accessed via the MassIVE repository (MSV000089657 and MSV000090218).

## Introduction

Glycosylation is one of the most common post-translational modifications (PTMs) of proteins, and it is of crucial importance since glycoproteins regulate several biological processes and cellular events.^1^ The past decades witnessed various improvements in separation science, mass spectrometric instrumentation, and data evaluation solutions; as a result, mass spectrometry (MS) coupled to liquid chromatography (LC or nano-LC) has become an indispensable tool in glycoproteomics.^2−4^ The analysis of glycoproteins is still often challenging due to their typically low concentration, the heterogeneity of glycan structures, and lower ionization efficiency of glycopeptides compared to unmodified peptides.^2,4^

The characterization of glycosylation using tandem mass spectrometric techniques is usually performed through the study of intact glycopeptides produced by enzymatic digestion of glycoproteins. This approach provides the most detailed view on the modification site on the protein, on the composition of the attached glycan, and on the identity of the peptide/protein through a single LC–MS/MS measurement.^5^

The depth of information that can be extracted by mass spectrometry depends on the instrument type, the fragmentation technique used, and the bioinformatics tools applied. Since glycopeptides have a diverse bonding pattern with considerably different bond strengths, complementary fragmentation techniques and/or multiple sets of experimental parameters are usually required for complete structural characterization.^4,6^ Among the diverse techniques,^7^ beam-type collision induced dissociation (CID) is the most widespread fragmentation in bottom-up proteomics, which can be operated on quadrupole time of flight (QTof) and Orbitrap instruments as well (in the latter case, also called higher-energy collisional dissociation, HCD).^8,9^ Depending on the collision energy (CE) setting, this method provides b- and y-type peptide sequencing ions, enabling peptide identification, or diagnostic glycan fragments with information on the oligosaccharide structure. At lower CE values, the glycan moiety is selectively cleaved, while at higher CE, the whole glycan part leaves, and the peptide backbone dissociates.^3−5,10−13^ In line with this, various works pointed out the benefit of the use of multiple CE values (stepped CE methods).^11,12,14−17^ Notable alternatives are electron transfer dissociation (ETD) and electron capture dissociation, which allow the site of modification to be deduced and are therefore particularly significant for O-glycopeptides lacking a consensus sequence.^5,18−20^ Combined methods (e.g., electron transfer/collision induced dissociation, ETciD, and electron transfer/higher-energy collisional dissociation, EThcD) are also emerging.^3,20,21^ Nevertheless, a recent systematic study indicated the superiority of HCD and stepped HCD techniques over ETD/EThcD methods for N-glycopeptides.^16^

Numerous scientific works have recently addressed the optimal choice of CE in the MS/MS investigation of N-glycopeptides, which appears to be even more important than in the case of unmodified peptides. Some of the works compared a handful of collision energy settings, or various fragmentation methods, and reported overall performance in N-glycopeptide identification from complex samples without any peptide-specific analysis.^13−17^ Other authors mapped energy dependence of scores or individual fragmentation pathways in detail, focusing on a few selected N-glycopeptide structures.^11,12,22,23^ Other aspects such as the formation of peptide + GlcNAc ions for site localization or structure-specific glycan analysis were also studied as a function of the collision energy.^24−26^

For the best performance in N-glycopeptide identification, existing studies agree that it is worth applying stepped methods, where ions are fragmented at two or three different CE values, and product ions from the different dissociation steps are acquired in a single MS/MS spectrum. The accumulation time is frequently distributed equally between the CE settings, but, e.g., Hinneburg et al.’s pioneering study, carried out on a QTof instrument, worked with 80 and 20% for the higher and lower energy component, respectively.^12^ As a result, various, somewhat different optimal methods were reported.^27^ The obtained values are specific to the mass spectrometer used. In the case of Thermo Orbitrap instruments, an *m*/*z*-dependent normalized value is used (normalized collision energy, NCE), which is supposed to help in transferability of the settings. However, it was found that different members of the Thermo Orbitrap equipment series still differ even in NCE terms.^27^ The direct transfer and comparison of reported CE settings are even more difficult with QTof instruments, where “unnormalized” CE is applied explicitly.

Glycopeptide identification is carried out using computer programs, and the data evaluation software may have a large impact on the results, e.g., on the set of identified N-glycopeptides. A recent comparative study showed that the use of different search engine results in considerable team-to-team variation even if the same experimental data is evaluated.^28^ Byonic and Protein Prospector were found to be the best solutions, based on several gauges of quality covering sensitivity and identification accuracy. Byonic is the most widely accepted and used commercial software for glycopeptide identification.^29^ It has a peptide centric nature; first, it searches the peptide part and handles the glycan as a (sometimes very large) PTM. Its FDR calculation focuses on the correctness of peptide sequence. In contrast, a glycan-first search is used in the relatively new pGlyco software series.^30−32^ Further, pGlyco is the first method that has separate characterization for the glycan and peptide parts of glycopeptides, thus providing glycan- and peptide-level quality control. Unfortunately, it was not involved in the abovementioned study, therefore its investigation and comparison with the widespread Byonic search engine would be of high interest.

Given the differences in the peak picking and scoring algorithm of available search engines,^4,33,34^ one may expect somewhat different experimental parameters to be optimal when different data evaluation procedures are used. This was indeed confirmed for unmodified peptides^35^ but has not yet been explored for glycopeptides.

Considering all the above, we aimed to complement literature works by a systematic CID MS/MS investigation, unprecedented for N-glycopeptides, in which

- we obtain specific results for a large number of individual tryptic N-glycopeptides, covering various peptide carrier and glycan structures,
- we map the energy dependence of search engine scores in detail (i.e., we focus on the confidence of identification and use many different collision energy settings), and
- we compare results from two search engines, the most frequently used Byonic and the freely available pGlyco 3.0, to evaluate the difference between their behaviors.

This approach is expected to provide several benefits, as some of us discussed in a recent review for other related analytes.^27^ We further leveraged the resulting glycopeptide-level information to design optimized measurement strategies, confirming the importance of both fine-tuning itself and using a diverse set of N-glycopeptide species for this purpose. Finally, our study is the first to provide reference data based on well-chosen standard materials for the transfer of the optimized method between mass spectrometers, which can guide scientists in choosing optimal settings on their experimental platforms in a laboratory or in a pharmaceutical industrial setting.

## Experimental Section

### Chemical Reagents

Unless otherwise stated, reagents and consumables were from Sigma-Aldrich (Sigma-Aldrich Kft., Budapest, Hungary). RapiGest SF was purchased from Waters (Milford, MA), while trypsin/Lys-C mix and trypsin digestion enzymes were from Promega (Madison, WI). α-1 acid glycoprotein (AGP), fetuin, and transferrin glycoprotein standards were obtained from Sigma-Aldrich (Sigma-Aldrich Kft., Budapest, Hungary), and the HeLa tryptic digest standard was from Thermo Fisher Scientific (Waltham, MA). Monoclonal antibody sample trastuzumab (product name: Herceptin) was obtained from Gedeon Richter Plc.

### Solvent Exchange of mAb Samples

A few nmol (2–5 nmol) of the sample was dissolved in LC–MS-grade water, the solution was subject to solvent exchange using a Millipore-10 kDa centrifugal filter.^36^ The filters were rinsed by LC–MS water, and then the protein sample was added, completed to 200 μL using ammonium bicarbonate buffer solution (200 mM), and centrifuged for 15 min (13,500*g*, 4 °C). Three additional cycles were performed, the first two by 200 mM ammonium bicarbonate solution and a third one using 50 mM ammonium bicarbonate solution. The resulting mAb solution (ca. 30–40 μL/protein) was divided into aliquots of 1 nmol.

### Digestion

In the case of glycoprotein standards (i.e., AGP, fetuin, transferrin, and mAb), 1 nmol of sample was subjected to enzymatic digestion. Blood plasma was digested in aliquots of 30 μg (see Material S1). Briefly, denaturation of the samples was performed by Rapigest SF, the S–S bridges were reduced by dithiothreitol followed by alkylation using iodoacetamide in the dark. Then, the samples were digested first by the Lys-C/trypsin mixture (1 h) followed by digestion using trypsin (3 h). The appropriate pH was set using ammonium bicarbonate buffer solution. Digestion was quenched by the addition of formic acid. The digests of glycoprotein standards and that of mAb were divided to aliquots of 200 pmol and were dried in SpeedVac. From each sample, one aliquot was dissolved in the injection solvent (98% water, 2% acetonitrile, and 0.1% formic acid) prior to nano-LC–MS/MS analysis. A mixture of three glycoprotein standards was also prepared from the digests of AGP, fetuin, and transferrin. The blood plasma digest was dried in SpeedVac, and cleanup was performed using a C_18_ spin column (Thermo Fisher Scientific) in aliquots of 15 μg using a protocol based on the manufacturer’s recommendation. The resulting samples were again dried in SpeedVac.

### Acetone Precipitation

HeLa tryptic digest and blood plasma digest were subject to a simple and cheap acetone solvent precipitation method in aliquots of 1 μg.^37,38^ The samples were dissolved in 15 μL of water +1% formic acid, and then 150 μL ice-cold acetone was added. The solution was stored at −20 °C overnight, resulting in the formation of a pellet, which may contribute to increasing the ratio of glycopeptides. The sample was centrifuged at 12000*g* for 10 min. The supernatant containing most of the peptides was removed by pipetting. The pellet fraction was dried in SpeedVac and redissolved in the solvent (98% water, 2% acetonitrile, and 0.1% formic acid) prior to nano-LC–MS/MS analysis.

### Mass Spectrometric Measurements

Nano-LC–MS/MS studies of the digested glycoprotein standards, mAb digest, and complex protein samples were performed using our standard laboratory methods for glycoproteomics investigation (see Material S2) with varying MS/MS collision energy settings. Briefly, samples were subjected to nanoLC–MS/MS analysis using a Dionex Ultimate 3000 RSLC nanoLC coupled to a Bruker Maxis II ETD Q-TOF via a CaptiveSpray nanoBooster ionization source operated in positive mode. (Glyco)peptides were separated on an Acquity M-Class BEH130 C18 analytical column using gradient elution following trapping on an Acclaim PepMap100 C18 trap column. Solvent A consisted of water + 0.1% formic acid, while solvent B was acetonitrile + 0.1% formic acid. Spectra were collected using a fix cycle time of 2.5 s and the following scan speeds: MS spectra at 2 Hz, CID on precursors at 4 Hz for abundant ones and at 0.5 Hz for peaks of low abundance. An active exclusion of 2 min after one spectrum was used except if the intensity of the precursor was elevated threefold. The use of exclusion is typical in mass spectrometric-based proteomics measurements; our respective settings are the typical values of Bruker instruments.

### Mass Spectrometric Experimental Series

**Figure 1:**
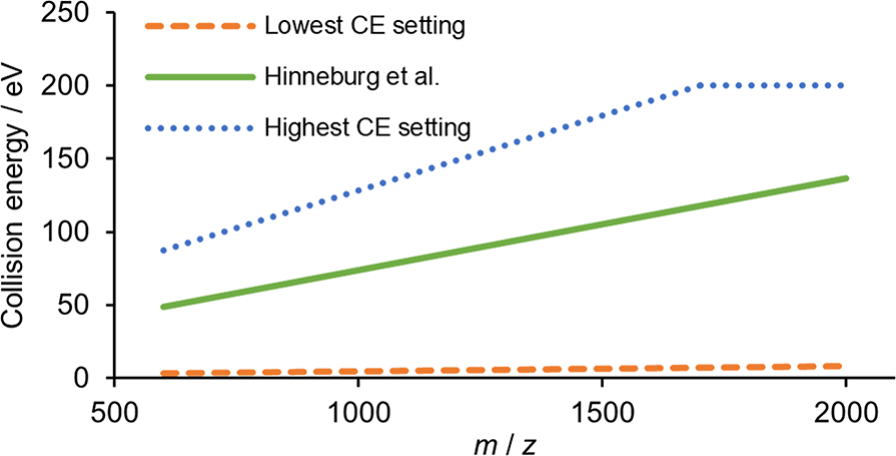
CE energy range, which was covered with the high CE component during the CE optimization process. The low energy component/high energy component ratio was set at 0.5, and high CE was applied in 80% of the fragmentation time.

Typically, CE values linearly dependent on precursor *m*/*z* are used, which takes into account the size of the species. In line with this, an *m*/*z*-dependent collision energy was employed in all of our experiments. Since several studies pointed out that the use of the stepped CE method is beneficial for the investigation of N-glycopeptides, we applied stepped CE setting involving two CE values referred as “high CE” and “low CE”. Our starting method for optimization, matching the setting published by Hinneburg et al., involved a high CE of 55 eV at 600 *m*/*z* and 135 eV at 2000 *m*/*z*, with a linear interpolation between the two values. On an Orbitrap, this corresponds to 43–49% NCE depending on the *m*/*z* value.^39,40^ The low CE component was set at half of the high CE, and the high CE condition was applied in 80% of the acquisition time. In the present study, we acquired several LC–MS/MS series with various fragmentation conditions. The value of high CE component, the low CE/high CE ratio, and the fraction of time spent on high CE were all varied. The details of the experimental series are summarized in Table 1 (see also Figure 1, and Supporting Information, Table S1).

**Table 1:**
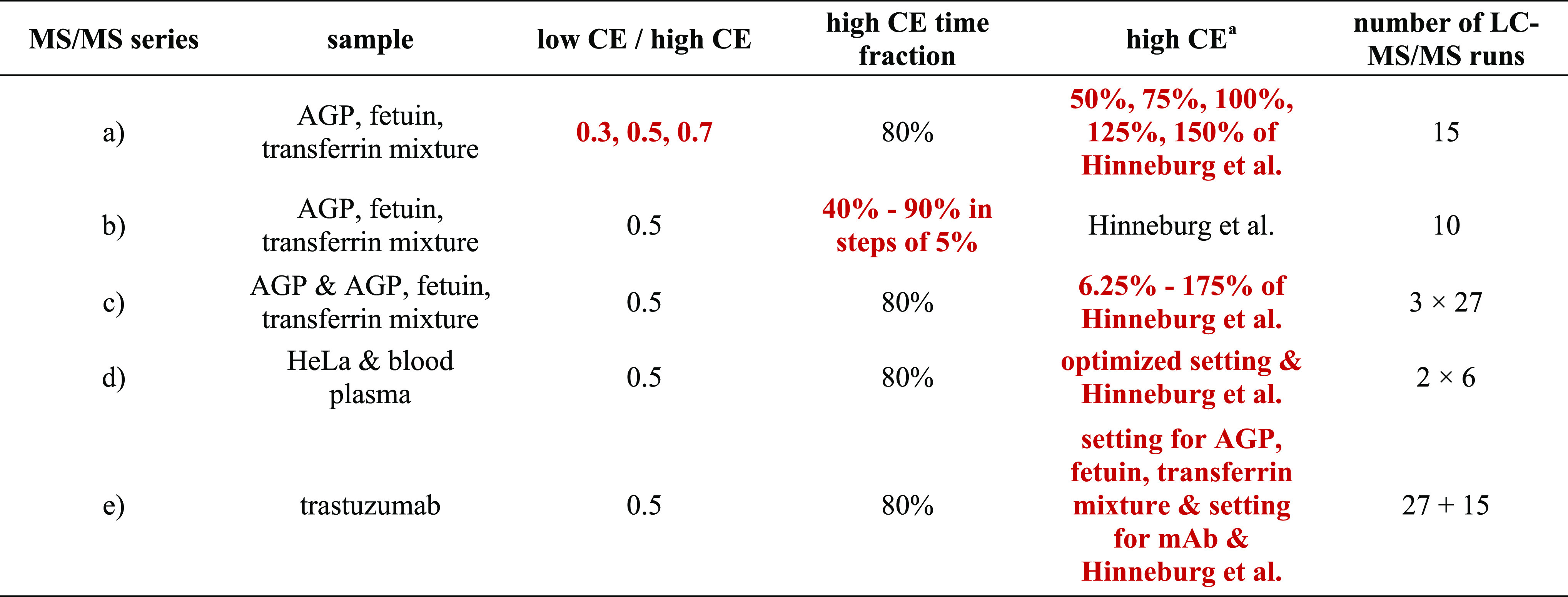
List of Experimental Nano-LC–MS/MS Series^b^

a 100% setting means 55 eV at 600 *m*/*z* and 135 eV at 2000 *m*/*z* with a linear interpolation between the two *m*/*z* values. This equals Hinneburg et al.’s setting. On an Orbitrap, this corresponds to 43–49% NCE depending on the *m*/*z* value (for more information, see Table S1).

b Parameters in burgundy were varied. In all experimental series, we used the stepped CE method with two CE values: a higher energy component (“high CE”) and a lower energy component (“low CE”). The “low CE/high CE” refers to the ratio of the two component, e.g., 0.5 means that the lower energy component is half of the higher energy component. The MS/MS acquisition time was distributed between the two components, and “high CE time fraction” refers to the fraction of fragmentation time allocated to the higher energy component. Further, in all measurements, we employed *m*/*z* dependent collision energy. Our starting point for optimization referred to as 100% was 50 eV at *m*/*z* 600 and 135 eV at *m*/*z* 2000 as a high CE component with a linear interpolation between the two *m*/*z* values. This setting equals to the method published by Hinneburg et al. More detailed information can be found in the SI (Table S1).

#### Ratio of the Lower and Higher Energy Component (a)

Tests were done on the mixture of AGP, fetuin, and transferrin digests using 2.2 pmol from all glycoproteins in each run. Five different high CE settings were used, which were 50, 75, 100, 125, and 150% of the original method of Hinneburg et al. These were combined with three different low CE/high CE ratios, 0.3, 0.5, and 0.7, resulting in 15 different MS/MS settings.

#### Time Distribution (b)

Tests were performed on the mixture of AGP, fetuin, and transferrin digests using 2.2 pmol from all glycoproteins in each run. The fraction of the MS/MS acquisition time allocated to the higher energy condition was systematically varied from 40 to 90% in steps of 5% resulting in 10 different MS/MS methods.

#### Detailed Stepped CE Dependence (c)

Detailed energy dependence investigations were carried out on AGP digest using 2.2 pmol glycoprotein per run and on the mixture of AGP, fetuin, and transferrin digests, injecting 2.2 pmol from all glycoproteins in each run. Stepped CE settings were applied with 80% of the time allocated to the higher energy component, and the low CE/high CE ratio was set to 0.5. The CE was systematically varied from 6.25 to 175% of the Hinneburg et al.’s setting (see Figure 1, and Supporting Information, Table S1) in steps of 6.25%, resulting in 27 different nano-LC–MS/MS runs. Experiments were performed with the use of inclusion lists based on DDA measurements taken with Hinneburg et al.’s CE method. Two lists for the mixture and one list for AGP were created.

#### Performance Check (d)

The above energy dependence studies allowed optimum energies to be determined for each individual N-glycopeptide, but obviously, we cannot directly apply these in practice since the identity of the glycopeptides is not known at the time of the measurement. The results on individual glycopeptides showed reasonably good *m*/*z*-dependent linear trends for the optimum energies for both Byonic and pGlyco search engines, and these formed the basis for CE choice in a practical DDA measurement run. We explored the potential gain via CE optimization by comparing the number of hits using Hinneburg et al.’s literature CE setting and our optimized MS/MS method in actual measurements. HeLa digest and blood plasma digest were used. The pellet fractions of acetone precipitation were investigated with injection amounts of 750 ng and 1.5 μg in the case of HeLa and blood plasma, respectively. Three repetitions were carried out with each CE setting, and data were evaluated using both Byonic and pGlyco search engines.

#### Measurements on mAb Samples (e)

Nano-LC–MS/MS experiments were performed on an mAb sample using 2 pmol tryptic digest in each run. First, the energy dependence study was carried out analogously to the mixture of the three glycoprotein digests and AGP tryptic digests as described above involving 27 nano-LC–MS/MS measurements. Then, based on the energy dependence, an optimal CE method was designed for the mAb N-glycopeptides. The CE method of Hinneburg et al., the CE setting optimized for all the N-glycopeptides of the glycoprotein mixture and AGP samples, and the CE optimized for the mAb N-glycopeptides were tested and compared in 5–5 repetitions (15 runs overall).

### Data Analysis

The raw QTof data were first recalibrated using Bruker Compass DataAnalysis software 4.3 (Bruker Daltonik GmbH, Bremen, Germany) for the internal calibrant. MS/MS spectra were searched against the appropriate protein database using Byonic v4.2.10 (Protein Metrics, Cupertino, CA)^29^ and pGlyco 3.0^31^ search engines. The measurements of glycoprotein mixture and AGP were evaluated using the amino acid sequences of the three glycoprotein standards (obtained from UniProt, December 2019); human SwissProt (November 2020) database was applied for the analysis of HeLa and blood plasma experiments, while the amino acid sequence of the mAb (obtained from DrugBank database, December 2018) was used for mAb samples. Byonic searches were carried out with the human N-glycan database of 182 structures without multiple fucose as implemented in Byonic, while the pGlyco-N-Human.gdb was used for pGlyco searches. Trypsin was set as the enzyme; a maximum of two missed cleavages were allowed, and cysteine carbamidomethylation was selected as a fixed modification. Regarding mass tolerance values and the list of variable modifications, recommendations of the Preview module of Byonic were used. The Byonic Excel reports and pGlyco FDR-Pro.txt reports were the input files for data aggregation carried out by the Serac program^36^ in the energy-dependent studies (see Determination of Optimal CE Setting Using Serac). The practical glycoproteomics performance of nano-LC–MS/MS runs using different CE methods was characterized by the number of hits using the following filtering conditions: Byonic score > 200 and logProb >2 for Byonic and 1% FDR for pGlyco.

### Determination of Optimal CE Setting Using Serac

For the study of the energy dependence of N-glycopeptide fragmentation, we used our recently developed program called Serac.^36^ The program collected identification scores as a function of collision energy from the energy-dependent mass spectrometric data series for the Byonic and pGlyco search engines and determined the optimal collision energy. Serac first extracted the data from Byonic Excel reports and the FDR-Pro.txt output files of the pGlyco program. Then, the Serac program normalized the score vs CE setting functions by dividing all values with the maximum score for the given glycopeptide ion. Byonic score values and pGlyco total scores were investigated. To ensure that we draw conclusions on the basis of confident N-glycopeptide identifications, only species meeting certain minimum requirements were selected by Serac. First, depending on the chosen measure of identification confidence, the Serac program only considered an N-glycopeptide ion identified at a given CE setting if its Byonic score exceeded 100 or its pGlyco score was above 5. Further, a glycopeptide ion was only included in the energy dependence analysis if it was identified at least at six consecutive collision energy settings and for at least one collision energy it was found to have a Byonic score value above 300 (being a “good” score) or pGlyco total score above 15.

For each N-glycopeptide, the Serac program determined the optimum energy from the normalized score vs collision energy setting data sets by fitting Gaussian functions. The score cutoff, while important to avoid false identifications biasing our results, resulted in no data points at low scores; therefore, two additional points with zero score at CE settings of 0 and 300% were added to avoid erroneously wide peaks to be fitted. The nonlinear fits were carried out by Serac, and the corresponding plots were generated using the levmar^41^ and PGPLOT^42^ libraries through their Perl Data Language interfaces. The positions of the center of the Gaussian peaks were considered as optimal values.

## Results and Discussion

### Ratio of Low Energy and High Energy Component of Stepped Collision Energy Setting and Time Fraction of High Energy Components

There is consensus in the literature that the MS/MS analysis of N-glycopeptides benefits from using stepped CE methods. We therefore decided to stick to the combination of two different CE values and begin our study with systematically investigating (1) the effect of the ratio of the collision energy of the higher and lower energy components of the stepped CE method and (2) the relevance of the fraction of the fragmentation time allocated to the lower and higher energy settings. With regard to the first issue, we carried out a nano-LC–MS/MS experimental series of 15 measurements with five different high energy choices combined with three different low CE/high CE ratios. The investigations were done from the digests of three standard glycoprotein mixtures, and data evaluation was performed using Byonic and pGlyco search engines. The ratio had only a slight influence on the number of successfully identified N-glycopeptides for both engines. The low CE/high CE ratio of 1/2 slightly outperformed the other values (0.3 and 0.7); therefore, we used this value during subsequent analysis (see Figures S1 and S2). Next, a series of 10 nano-LC–MS/MS experiments were performed, varying the MS/MS acquisition time distribution between the high and low CE component. Overall, we found that there is a broad plateau in the number of identifications for both search engines as the time spent under the high CE condition is varied from 50 to 80%. Byonic data analysis showed optimum results at 80–90%, reflecting the peptide-centric nature of this search engine, while using pGlyco, a maximum around 50–70% appears, in line with higher focus on the glycan structure (see Figures S3 and S4). Considering these findings, we kept the choice of Hinneburg et al.^12^ throughout the project, specifically, using the high CE value for 80% of the acquisition time. We note in passing that using three instead of two different CE values in a stepped method did not bring any further improvement in our experiments (see later for the details).

We furthermore note here that pGlyco tends to identify a smaller number of glycopeptides. Further analysis of the exact reason would be beyond the scope of the present work, but it is well demonstrated in the literature that search engines using fundamentally different algorithms for identification and scoring can produce notably different results even when applied to the same experimental data.^28^ For example, the specific types of fragments they look for and their relative importance in the scoring are a crucial aspect; Byonic appears to be more peptide-focused, so we may speculate that pGlyco might be stricter in accepting a certain match if the spectrum contains less information about the glycan part.

### Collision Energy Dependence for Individual N-Glycopeptides

Having settled the key experimental parameters, we then moved on to examine the detailed collision energy dependence of identification score of N-glycopeptides to determine optimal CE settings for various N-glycopeptide species. Experiments were taken on the AGP tryptic digest and mixture of the three glycoprotein standard digests. To ensure that a given N-glycopeptide is measured at all (most of) the CE settings, inclusion lists were determined from preceding DDA experiments. Based on our preliminary investigations on the CE ratio and time fraction, we used the stepped method proposed by Hinneburg et al. as a starting setting. Then, we increased and decreased the CE value in the steps of 6.25%, and then we created overall 27 nano-LC–MS/MS methods mapping the CE range from 6.25 to 175%. The largest value that can be set at our instrument is 200 eV; therefore, an upper limit was used at this value (see Figure 1).

We constructed energy dependence curves of Byonic scores and pGlyco total scores for N-glycopeptides. When a given species was identified more than once in the same LC–MS/MS run, that is, measured several times at the same CE setting, the best scoring match was accepted. Overall, we identified 227 and 199 N-glycopeptides using the Byonic and pGlyco search engines, respectively. Among these, 196 and 127 were considered sufficiently reliable to be evaluated in the energy-dependent analysis (see Data Analysis). The investigated species covered 15 and 14 different peptide backbones combined with 26 and 19 different oligosaccharide structures for Byonic and pGlyco search engines, respectively. Practically, the N-glycopeptides analyzed by pGlyco software were a subset of those examined by Byonic program; there was only one glycoform, which appeared only in the pGlyco data set.

**Figure 2:**
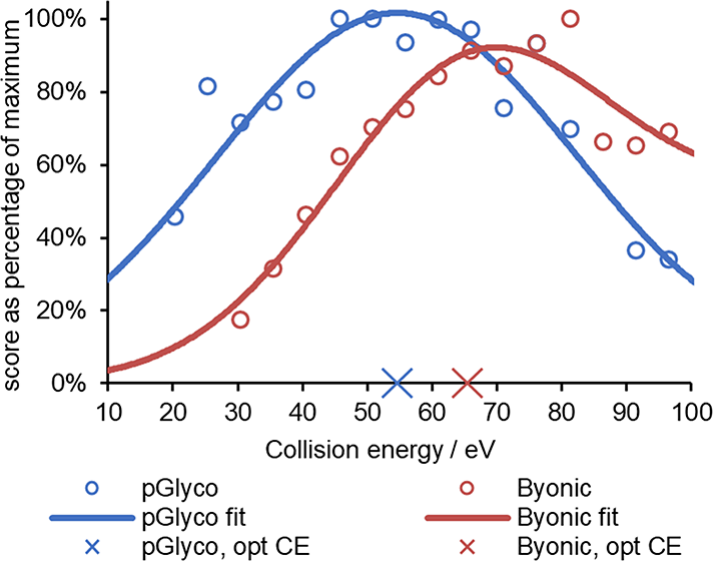
Result of fitting Gaussians to the energy dependence data points (score as % of the maximum value vs higher component of the stepped collision energy in eV) of the QDQCIYNTTYLNVQR-HexNAc(6)Hex(7)Fuc(1)NeuAc(4)^5+^ N-glycopeptide evaluated with Byonic and pGlyco search engines. Symbols denote measured data, while solid lines depict the model functions. The peak positions of the latter are marked by crosses on the horizontal axis. Burgundy circles and blue circles depict Byonic and pGlyco results, respectively.

Figure 2 depicts representative examples of the experimental points together with the fitted Gaussian functions for QDQCIYNTTYLNVQR-HexNAc(6)Hex(7)Fuc(1)NeuAc(4)^5+^ N-glycopeptide (derived from AGP). The higher component of the stepped CE method is shown on the *X* axis. The centers of the Gaussian functions were accepted as collision energy optimum values; they are denoted by crosses on the horizontal axis. The resulting optima are 65.6 and 54.6 eV for Byonic and pGlyco, respectively. As it was mentioned earlier, MS/MS spectra of N-glycopeptides show various types of product ions including glycoform-specific oxonium ions, B- and Y-type glycan and glycopeptide fragments, and b/y-type peptide fragments. We think that the key reason for the different optima is that the two different search engines look for and utilize the various types of fragment ions in a different manner.

### Trends in Optimal Collision Energies

**Figure 3:**
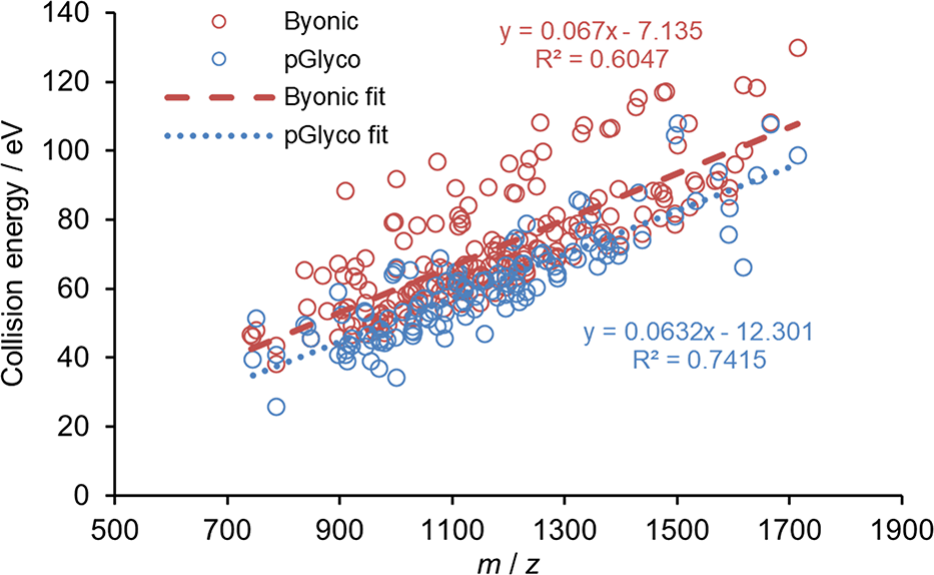
Higher component of the optimal collision energies in eV as a function of *m*/*z*. Burgundy and blue circles indicate the optimum higher energy component using Byonic and pGlyco search engines, respectively. Dashed and dotted lines represent linear fits of the measured data.

Figure 2 already anticipates that the optimal CE setting might be somewhat lower for pGlyco than for the Byonic search engine. Indeed, this trend is general. We plotted the optimum collision energies (more precisely, the higher component) as a function of the N-glycopeptide ion *m*/*z* value (see Figure 3). Peak positions of the fits are represented by burgundy and blue circles belonging to the Byonic and pGlyco optima, respectively. Apparently, the determined optima follow linear trends with respect to *m*/*z* with relatively large *R*^2^ values (see dashed/dotted lines). It can be seen that pGlyco has a trend line at ca. 5–10 eV lower setting than the Byonic search engine, indicating that the difference in search engine may have influence on the whole trend itself.

**Figure 4:**
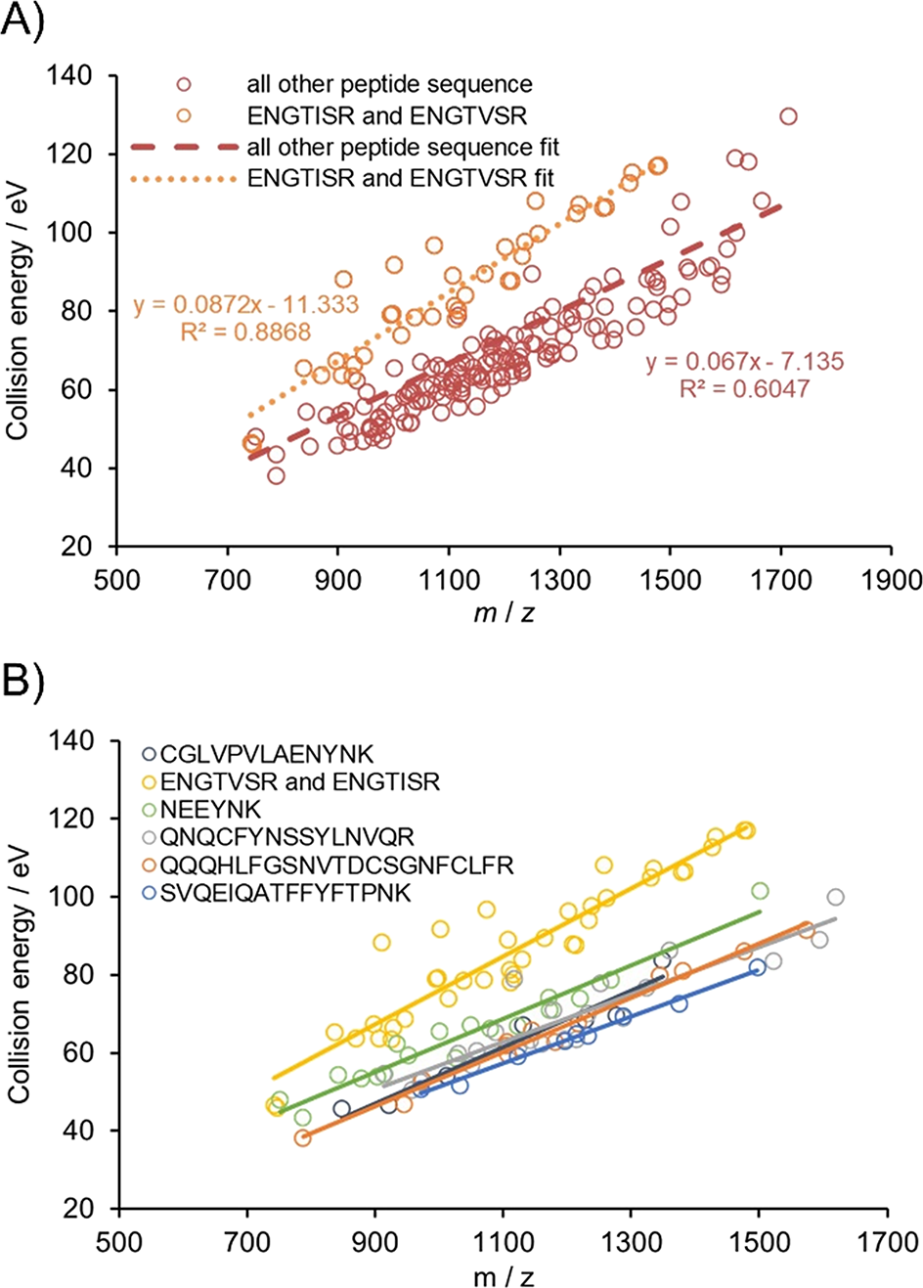
Influence of amino acid sequence on the optimal CE. (A) Higher component of the optimal collision energies in eV as a function of *m*/*z* using the Byonic search engine. Orange circles indicate the optimum higher energy component for N-glycopeptides with the ENGTISR or ENGTVSR peptide backbone, while burgundy circles belong to the positions of all the other N-glycopeptide species. Dashed and dotted lines belong to the linear fits of the measured data points. (B) Higher component of the optimal collision energies in eV as a function of *m*/*z* using the Byonic search engine for N-glycopeptides having six different amino acid sequences. Different colors belong to the different peptide backbones. Solid lines represent the linear fits to the experimental points and emphasize the separate linear trends for the various sequences.

A closer examination of our data reveals that the amino acid sequence is a major influencing factor. As an example, Figure 4A depicts our obtained optima for Byonic, and all the points belonging to glycopeptides with ENGTISR or ENGTVSR peptide backbone (derived from AGP) are marked by orange. Results corresponding to these peptides lie most distant from the trend line corresponding to all studied compounds. In general, N-glycopeptides sharing peptide sequences follow nice but distinct linear trends (see Figure 4B with a few more examples). A similar phenomenon is also present for the pGlyco data (see Figure S5), although the difference is somewhat less prominent.

### Comparison to Literature Results

**Figure 5:**
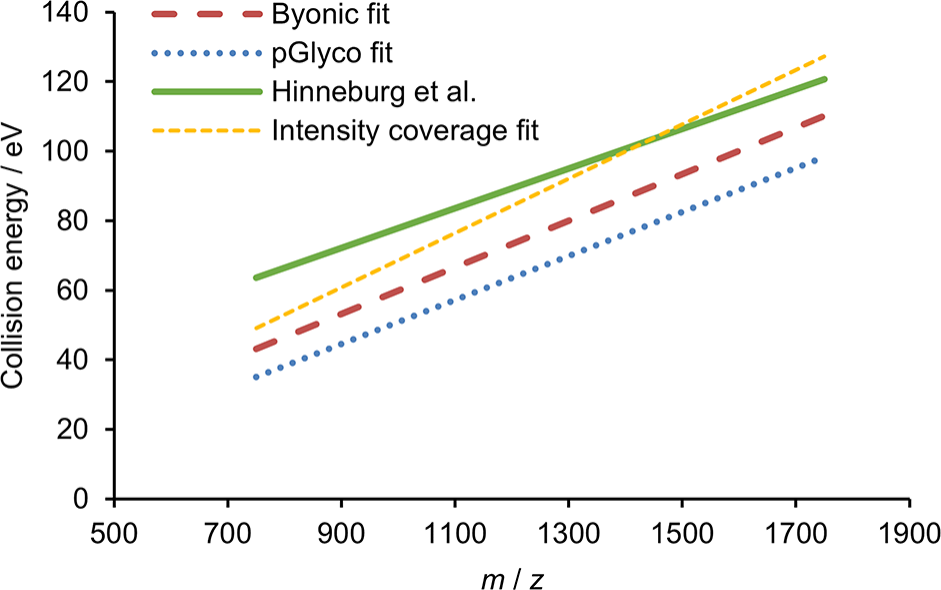
Higher component of the optimal collision energies in eV as a function of *m*/*z* with various data evaluation methods: using Byonic (burgundy) and pGlyco (blue) search engines as well as maximizing the intensity coverage in spectra identified by a combination of Mascot and GlycoQuest (yellow). Optimal energies for the peptide backbone from Hinneburg et al. are also shown (green).

How do our linear fits of optimal collision energies as a function of *m*/*z* compare to those of Hinneburg et al.?^12^ As apparent from Figure 5, our Byonic and pGlyco results both fall notably below the line corresponding to their method, with 15–25 eV lower CE values being optimal. The search engine dependence may play a key role in this difference since they used another approach, based on the Mascot and GlycoQuest search engines, with the peptide intensity coverage as a measure of the identification confidence. To assess the impact of the data analysis, we implemented their approach on our experimental data, though we do not expect to exactly reproduce their results, based on single collision energies, with our stepped methods. Our obtained trend line shown on Figure 5 (“intensity coverage fit”, yellow) highlights the significant impact of the evaluation method but also underlines the role of further factors.

Notably, Hinneburg et al. examined mainly synthetic N-glycopeptides, all having the same amino acid composition.^12^ In contrast, the present study uses a much broader selection of N-glycopeptides, covering various peptide backbones differing in length, amino acid composition, etc. Figure 4 confirms that various peptide structures are needed for general optimization because the use of only one sequence may lead to biased results.

In addition, though both studies used a QTof mass spectrometer from Bruker, instrumental differences may also contribute, as we have seen in the past for closely related Orbitrap instruments.^27^ Different internal energy distribution on the two instruments, caused by differences of the ESI source (e.g., cone voltage and gas pressures) or voltages applied during ion transfer might be relevant candidates.^43−45^

We note here that the different collision energy selection methods actually lead to perceivable differences at the level of individual peptides. This is illustrated by a few selected MS/MS spectra for three different N-glycopeptides taken at the CE setting of Hinneburg et al. and our optimal CE setting; these are presented in Figures S6–S8.

### Comparison to Unmodified Peptides

**Figure 6:**
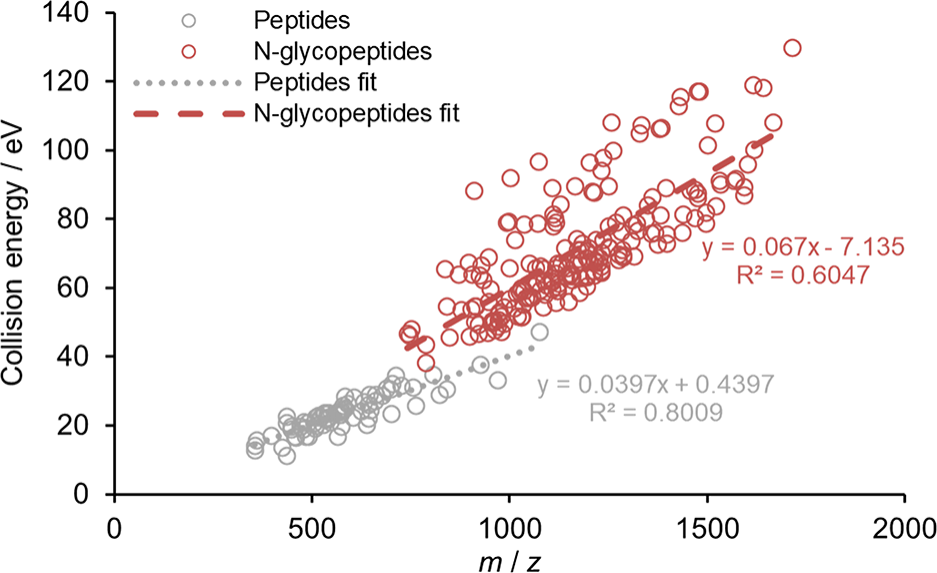
Optimal collision energies in eV as a function of *m*/*z* using the Byonic search engine. Burgundy circles indicate the optimum higher energy component for N-glycopeptides, while gray circles depict the optimum ones for unmodified peptides. Dashed and dotted lines represent the linear fits of the measured data points.

As discussed, N-glycopeptides are measured using a stepped collision energy setting, where the low energy component serves to produce glycan fragments, while the high energy component is supposed to produce peptide backbone fragments. Therefore, it appears meaningful to compare the higher components of the obtained CE settings with the single CE values determined for unmodified tryptic peptides of HeLa digest (see Figure 6). The latter were measured on the same instrument earlier in our group.^35^ Since pGlyco is designed for glycopeptide identification only, no pGlyco analysis was carried out in this section. It was found that N-glycopeptides need ca. 30–50% more CE (higher component) than peptides, meaning that they require ca. 30–50% more internal energy to produce peptide sequencing b/y-type ions. This can be explained by the huge size and labile nature of the attached glycan, which takes away a large amount of energy upon dissociation. Therefore, less energy remains for the peptide backbone fragmentation, which is typically produced in consecutive dissociation processes.

### Performance of Optimized Energy Setting

The optimal CE settings of individual glycopeptides follow reasonably good *m*/*z*-dependent linear trends, and we used this relationship to form the basis of optimal CE choice in practical DDA measurements. The results of the two search engines are relatively close to each other, and both trend lines lie below the setting published by Hinneburg et al.; further, Byonic is much more frequently used in the scientific community than pGlyco. Therefore, we created an “optimized MS/MS method” using the linear fit of the Byonic optima (see Figure 3) and compared it to the “Hinneburg et al. MS/MS method”. Three repeated nano-LC–MS/MS measurements were recorded with both methods. The pellet fractions of acetone precipitation of HeLa digest and blood plasma digest were used as samples.

**Figure 7:**
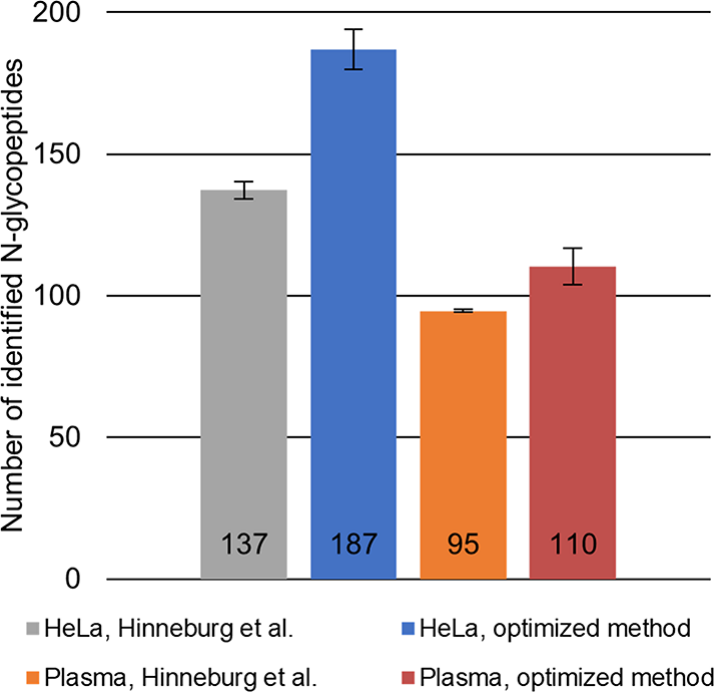
Number of identified unique N-glycopeptides as an average of three repeats analyzed by the Byonic search engine. Error bars indicate ±1 standard deviation.

The performance of the CE settings was characterized by the number of successfully identified N-glycopeptides (see the Data Analysis section for the identification criteria). N-glycopeptides identified in two or more charge states were regarded as one hit, and the values from the three repetitions were averaged. Figure 7 illustrates the results as a bar chart for the Byonic search engine. As it can be seen, a significant increase could be achieved using the optimized experimental setting. The results and trends are analogous for pGlyco, although significantly fewer hits were obtained, and the optimization counts more (see Figure S9). The smaller number of N-glycopeptide detection is in agreement with a recent preprint comparing algorithms and therefore corroborates their results.^46^

In addition to the number of identifications, their confidence also increased, as reflected by the score and logProb values averaged over the N-glycopeptides found by both the optimized and Hinneburg et al.’s methods. In the case of the Byonic search engine, both the average score and average logProb values increased significantly upon optimization of the CE settings. Namely, the average Byonic score increased to 358 from 314 and to 356 from 324 in the case of HeLa and plasma samples, respectively. The average logProb changed to 6.41 from 6.25 for HeLa measurement and to 6.68 from 6.02 for the blood plasma sample. Data evaluation with pGlyco showed that the average glycan score was larger ca. by a factor of two for the optimized MS/MS measurements. More precisely, the average glycan score increased to 35 from 17 and to 56 from 27 in the case of HeLa and plasma samples, respectively. The average peptide score somewhat decreased or did not change, resulting in moderate increase in the average total score value.

Though the use of two energies for glycopeptide analysis follows logically from the two significantly different types of bonds to be fragmented, the use of three different energies is also widespread in the literature. In their analysis, Yang et al. highlighted that an additional energy step between those optimal for peptide and glycan fragments is highly beneficial for the formation of b/y + monosaccharide ions.^14^ These ions are particularly important for glycosylation site localization, but as noted by Riley et al., site localization is of minor importance for N-glycosylation as the tryptic glycopeptides rarely contain more than one consensus sequence.^16^ We therefore did not expect much benefit in our experiments, but we did test the impact by adding a third CE step at the midpoint between the high and low energy levels. Indeed, neither the number of hits nor the average score showed improvement over our two-energy optimized method (see Table S2).

### Application to an mAb Sample

The identification of oligosaccharide structures and characterization of N-glycosylation patterns are highly relevant but still challenging task for protein biotherapeutics.^47^ Therefore, we tested our approach on a monoclonal antibody as well. First, we carried out CE optimization on an mAb sample analogously to the previous optimization process on the glycoprotein standards. In mAbs, there is a single N-glycosylation site on the tryptic peptide EEQYNSTYR. Therefore, an energy-dependent LC–MS/MS experimental series was acquired for the mAb sample, and optimal CE settings for N-glycopeptides containing this site were determined. We found that an mAb-specific optimization produced parameters very similar to that of based on the mixture of three glycoprotein standards.

Further, performance comparison of various CE settings was carried out. LC–MS/MS measurements were performed with three different CE methods: optimized method for glycoprotein standards, optimized method for mAb samples, and Hinneburg et al.’s method (see Table 1). Though the small number of mAb N-glycopeptides makes statistically sound conclusions difficult, improvements of 10–30% are typically seen in number of identifications and average score values for both the Byonic and pGlyco search engines (see Table S3).

### Practical Guide for Transferability

So far, we have demonstrated that an optimized CE setup is highly advantageous for the identification of N-glycopeptides. Our experience shows that the transferability of optimized CE parameters between instruments is somewhat limited but redetermining the trend line and the associated optimal settings on another instrument via the investigation of the same large set of species would be admittedly very cumbersome.

Instead, we propose that measurement of a few, carefully selected N-glycopeptides, representative of the trend line, could be used as a streamlined approach to quickly obtain the optimized parameters on another instrument. We recommended and successfully applied this concept for tryptic peptides earlier.^35^ The idea is that even though the trend line itself may differ between instruments, the property of whether a given species lies close to the trend line or farther away seems well conserved. We have therefore collected a list of reference N-glycopeptides, for which the optimum CE was close to (within 8% of) the respective trend line in our experiments for both investigated search engines. To fine-tune another instrument, a set of measurements with varying CE is still needed, but it is sufficient to focus on this small set of N-glycopeptides easily obtainable from standard glycoproteins (AGP, fetuin, and transferrin). Since these species cover the full *m*/*z* range and their optima are expected to lie close to the trend line determined on a much larger set, the optimal settings can be obtained by fitting a line to the optima of only this limited set of species. The proposed set of species is provided in the SI (see Table S4). Note that even measuring all these glycopeptides is not strictly necessary, five to six data points might be sufficient for fine-tuning the CE for a particular instrument. Such a clean and straightforward protocol, based on qualified reference materials, which may be easily available in the case of glycopeptide standards, can expectedly meet the requirements for the transfer of different analytical methods in the pharmaceutical sector as well.

The present study was performed on a QTof instrument, but the proposed fine-tuning protocol can be transferred to any Orbitrap mass spectrometers as well. Earlier studies on peptides showed that a few eV adjustment of the collision energy results in MS/MS spectra nearly identical using CID or HCD in a wide energy range.^9^ Further, the energy dependence of peptide identification confidence shows comparable trends. Our optimized CE values for QTof can be used as starting settings for further fine-tuning using the conversion between eV and NCE%:

collision energy (eV) = NCE (%) × (precursor *m*/*z*)/500 × (charge factor)^39,40^

The charge factor equals to 0.9, 0.85, 0.8, and 0.75 for species having 2+, 3+, 4+, and 5+ charges, respectively.

## Conclusions

The characterization and identification of N-glycopeptides are usually based on MS/MS measurements, for which the CE setting is of key importance. Our aim was to determine the optimal choice for a large set of N-glycopeptides covering various peptide backbones and numerous different glycan structures. Several nano-LC–MS/MS experimental series were carried out on commercially available glycoprotein standards. Data evaluation was performed using both the widely used Byonic search engine and the pGlyco program. Based on the results on individual N-glycopeptides, we designed an actual CE setup and tested its performance over Hinneburg et al.’s recommendations using complex biological samples and mAb sample. The main conclusions that can be drawn from our investigations are the following:

- While the optimum energies for N-glycopeptides follow a discernible *m*/*z*-dependent linear trend, individual species show a rather large variation. It was found that one of the main factors influencing the optimum is the amino acid sequence. To our knowledge, ours is the first study to clearly demonstrate this impact and to highlight that a generic optimization process should include species with various peptide backbones.
- N-glycopeptides need ca. 30–50% more CE than unmodified peptides to generate peptide sequencing b- and y-type ions. This can be explained by the fact that upon CID, N-glycopeptides lose the glycan part first, and peptide fragments are produced via consecutive fragmentation processes. The leaving oligosaccharide moiety takes away a huge amount of energy.
- Based on results on individual N-glycopeptides, we designed an experimental CE setup. Our proposed optimal method for our instrument and the studied search engines encompasses lower energies than those published by Hinneburg et al., but for our workflow, it resulted in the identification of 15–50% more glycopeptides from HeLa and blood plasma samples, as compared to the previously recommended setting. Further, the confidence of the hits is also increased, as characterized by the score values. These findings clearly point out that instrument specific fine-tuning, potentially taking into account the search engine as well, is beneficial. Application on a monoclonal antibody sample also showed improvements.
- We proposed a fine-tuning protocol involving the measurement of only few, adequately selected reference N-glycopeptides from the digest of commercially available glycoprotein standards. It can provide parameters close to those optimized using several hundreds of N-glycopeptide species.

Our results clearly demonstrate the benefit of targeted collision energy optimization for the specific analytical requirements of N-glycopeptides and the diversity of N-glycopeptide behavior that needs to be taken into account in such an optimization. While 70% of Hinneburg et al.’s values might be a good starting point, we proposed a protocol that makes the fully optimized results easily available to scientists wanting to set up their mass spectrometric platforms. Further studies to help exploit the potential in full proteomics workflows are ongoing in our laboratory.

## Supporting Information Available

(Material S1) Details of enzymatic digestion, (Material S2) details of nano-LC–MS/MS measurements, (Table S1) details of MS/MS CE settings of the energy-dependent studies, (Figure S1) ratio of low energy and high energy component (Byonic), (Figure S2) ratio of low energy and high energy component (pGlyco), (Figure S3) time fraction of high energy component (Byonic), (Figure S4) time fraction of high energy component (pGlyco), (Figure S5) higher energy component of the optimal CE setting for N-glycopeptides with ENGTISR and ENGTVSR peptide backbones analyzed by pGlyco, (Figure S6) example MS/MS spectra of VVHAVEVALATFNAESNGSYLQLVEISR-HexNAc(5)Hex(6)NeuAc(3)^5+^, (Figure S7) example MS/MS spectra of SVQEIQATFFYFTPNK-HexNAc(5)Hex(6)NeuAc(3)^4+^, (Figure S8) example MS/MS spectra of CGLVPVLAENYNK-HexNAc(4)Hex(5)NeuAc(1)^4+^, (Figure S9) performance of optimized setting analyzed by pGlyco, (Table S2) impact of using three collision energy steps on the performance of glycopeptide analysis, (Table S3) results on mAb samples, and (Table S4) list of reference N-glycopeptides (PDF)
