# Supplementary material for: Diversity Matters: Optimal Collision Energies for Tandem Mass Spectrometric Analysis of a Large Set of N-Glycopeptides
Source: J Proteome Res. 2022 Oct 6;21(11):2743–53. doi: 10.1021/acs.jproteome.2c00519 (PMC9639208; doi:10.1021/acs.jproteome.2c00519)
Supplement: Supplementary file 1 — pr2c00519_si_001.pdf [file pr2c00519_si_001.pdf]

Diversity matters: Optimal collision energies for tandem mass spectrometric analysis of a large set of N-glycopeptides

Helga Hevér, Kinga Nagy, Andrea Xue, Simon Sugár, Kinga Komka, Károly Vékey, László Drahos, Ágnes Révész

Supporting Information

Table of Contents

Further experimental details

Material S1: Details of Enzymatic Digestion

Material S2: Details of Nano-LC-MS/MS Measurements

Details of MS/MS Collision Energy Settings

Table S1: Details of MS/MS CE Settings of the Energy Dependent Studies

Ratio of Low Energy and High Energy Component

Figure S1: Ratio of Low Energy and High Energy Component (Byonic)

Figure S2: Ratio of Low Energy and High Energy Component (pGlyco)

Time Fraction of High Energy Component

Figure S3: Time Fraction of High Energy Component (Byonic)

Figure S4: Time Fraction of High Energy Component (pGlyco)

Higher Energy Component of the Optimal CE Setting for N-glycopeptides with ENGTISR and ENGTVSR Peptide Backbone Analyzed by pGlyco

Figure S5: Higher Energy Component of the Optimal CE Setting for N-glycopeptides with ENGTISR and ENGTVSR Peptide Backbone Analyzed by pGlyco

Example MS/MS Spectra Taken at CE Setting of Hinneburg et al. and Optimal CE Setting

Figure S6: Example MS/MS Spectra of VVHAVEVALATFNAESNGSYLQLVEISR-HexNAc(5)Hex(6)NeuAc(3)$^{5+}$

Figure S7: Example MS/MS Spectra of SVQEIQATFFYFTPNK-HexNAc(5)Hex(6)NeuAc(3)$^{4+}$

Figure S8: Example MS/MS Spectra of CGLVPVLAENYNK-HexNAc(4)Hex(5)NeuAc(1)$^{4+}$

Performance of Optimized Setting Analyzed by pGlyco

Figure S9: Performance of Optimized Setting Analyzed by pGlyco;

Results with a 3-step method

Table S2: Impact of Using 3 Collision Energy Steps on the Performance of Glycopeptide Analysis

Results on mAb Samples

Table S3: Results on mAb Samples

List of Reference N-glycopeptides

Table S4: List of Reference N-glycopeptides

Further experimental details

Material S1. Details of Enzymatic Digestion

From glycoprotein standards and mAb sample, 1 nmol was transferred to LoBind Eppendorf tubes and completed to 25±5 µL using LC-MS water with MeOH reaching 5 % final concentration. From human blood plasma, 30 µg was used as aliquots analogously. Denaturation of the proteins and S–S bridge reduction were performed by adding Rapigest SF of 0.5 % (5 µL) and dithiothreitol of 200 mM (2 µL) and incubating in block heater at 60 °C for 30 min. The pH was set using 200 mM ammonium bicarbonate buffer solution (5 µL) followed by alkylation using 200 mM iodoacetamide (2.5 µL) in the dark at room temperature for 30 min. Then the samples were digested first by 1 µL 10 pmol/ µL Lys-C/Trypsin mixture at 37 °C for 60 min, followed by digestion using trypsin (1 µL, 40 pmol/µL) at 37 °C for 180 min in block heater. Digestion was quenched by the addition of 1.5 µL formic acid. Glycoprotein standards and mAb sample were divided to aliquots of 200 pmol and were dried in SpeedVac. at 50 °C. The blood plasma digest was dried in SpeedVac and cleanup was performed using C18 spin column (Thermo Fisher Scientific) in aliquots of 15 µg using a protocol based on manufacturer's recommendation. The resulting samples were again dried in SpeedVac. Each sample was dissolved in injection solvent (98% water, 2% acetonitrile and 0.1% formic acid) prior to nano-LC-MS/MS analysis.

Material S2. Details of Nano-LC-MS/MS Measurements

Liquid chromatography-mass spectrometry investigations were carried out on a Bruker Maxis II ETD Q-TOF (Bruker Daltonics, Bremen, Germany) mass spectrometer equipped with a CaptiveSpray nanoBooster ionization source coupled to an Ultimate 3000 NanoRSLC System (Dionex, Sunnyvale, CA, USA) under the control of Hystar v. 3.2 (Bruker Daltonics, Bremen, Germany). Sample digest was injected onto an Acclaim PepMap 100 C18 trap column (5 µm, 100 Å, 100 µm × 20 mm, Thermo Fisher Scientific, Waltham, MA, USA) using 0.1% trifluoroacetic acid (TFA). (Glyco)peptides were separated on an Acquity M-Class BEH130 C18 analytical column (1.7µm, 130 Å, 75µm x 250mm Waters, Milford, MA) at 48 °C using a flow rate of 300 nl/min. The gradient was as follows: 4% B from 0 to 11 min, followed by a 120 min gradient to 50% B, then the concentration of the solvent B was elevated to 90% in 1 min and kept there for 10 min; solvent A was 0.1% formic acid (FA) in water, while solvent B was 0.1% FA in acetonitrile.

Sample ionization was achieved in the positive electrospray ionization mode via a CaptiveSpray nanoBooster ion source. The capillary voltage was set to 1300 V, the nanoBooster pressure was 0.2 bar, the drying gas was heated to 150 °C, and the flow rate was 3 l/min. Internal mass calibration was performed via lock mass for each run using sodium formate according to Brukers' recommendation: 1 mmol sodium formate in 15% MeOH solution were infused at a flow rate of 0.03 ml/h into Acclaim PepMap 100 C18 trap column (5 µm, 100 Å, 100 µm × 20 mm, Thermo Fisher Scientific, Waltham, MA, USA). The ion transfer parameters were set as follows: prepulse storage 10 µs, quadrupole ion energy 5 eV, Funnel 1 RF 400 Vpp, Multipole RF 400 Vpp. The collision RF was set to 1200 Vpp, and the ion transfer time was 120 µs. For the MS measurements, a fix cycle time of 2.5 sec was used. MS spectra were acquired over a mass range of 150–3000 m/z at 2 Hz, while CID was performed at 4 Hz for abundant precursors and at 0.5 Hz for ones of low abundance.

Details of MS/MS Collision Energy Settings

Table S1. Details of MS/MS CE settings of the energy dependent nano-LC-MS/MS studies. In all experimental series, we used stepped CE method with two CE values: a higher energy component ("high CE") and a lower energy component ("low CE"). In all measurements, we employed m/z dependent collision energy. Our starting point for optimization referred to as 100% was 50 eV at m/z 600 and 135 eV at m/z 2000 as high CE component with a linear interpolation between the two m/z values. Note that the highest possible setting on our instrument is 200 eV, which we took into account in the setup, and which is reflected in the table. The "low CE / high CE" refers to the ratio of the two component, e.g., 0.5 means that the lower energy component is half of the higher energy component. The MS/MS acquisition time was distributed between the two components and "high CE time fraction" refers to the fraction of fragmentation time allocated to the higher energy component. On an Orbitrap instrument, our starting method corresponds to 43-49% NCE depending on the m/z value. Conversion between NCE% and eV can be carried out with the equation:

$$\text{collision energy (eV)} = \text{NCE (\%)} \times (\text{precursor } m/z) / 500 \times (\text{charge factor}).$$

The charge factor equals to 0.9, 0.85, 0.8 and 0.75 for species having 2+, 3+, 4+ and 5+ charges, respectively.

(see table on next page)

MS method	high CE m /z	high CE CE / eV	low CE / high CE	high CE time fraction
100.00%	600<br>2000	50.00<br>135.00	0.5	80%
12.50%	600<br>2000	6.25<br>16.88	0.5	80%
18.75%	600<br>2000	9.38<br>25.31	0.5	80%
25.00%	600<br>2000	12.50<br>33.75	0.5	80%
31.25%	600<br>2000	15.63<br>42.19	0.5	80%
37.50%	600<br>2000	18.75<br>50.63	0.5	80%
43.75%	600<br>2000	21.88<br>59.06	0.5	80%
50.00%	600<br>2000	25.00<br>67.50	0.5	80%
56.25%	600<br>2000	28.13<br>75.94	0.5	80%
62.50%	600<br>2000	31.25<br>84.38	0.5	80%
68.75%	600<br>2000	34.38<br>92.81	0.5	80%
75.00%	600<br>2000	37.50<br>101.25	0.5	80%
81.25%	600<br>2000	40.63<br>109.69	0.5	80%
87.50%	600<br>2000	43.75<br>118.13	0.5	80%
93.75%	600<br>2000	46.88<br>126.56	0.5	80%
100.00%	600<br>2000	50.00<br>135.00	0.5	80%
106.25%	600<br>2000	53.13<br>143.44	0.5	80%
112.50%	600<br>2000	56.25<br>151.88	0.5	80%
118.75%	600<br>2000	59.38<br>160.31	0.5	80%
125.00%	600<br>2000	62.50<br>168.75	0.5	80%
131.25%	600<br>2000	65.63<br>177.19	0.5	80%
137.50%	600<br>2000	68.75<br>185.63	0.5	80%
143.75%	600<br>2000	71.88<br>194.06	0.5	80%
150.00%	600<br>2000	75.00<br>200.00	0.5	80%
156.25%	600<br>1885	78.13<br>200.00	0.5	80%
162.50%	600<br>1800	81.25<br>200.00	0.5	80%
168.75%	600<br>1729	84.38<br>200.00	0.5	80%
175.00%	600<br>1700	87.50<br>200.00	0.5	80%

Ratio of Low Energy and High Energy Component

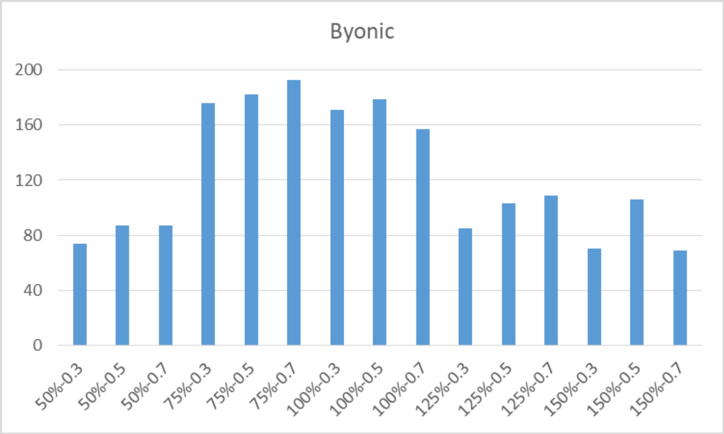

Fig. S1 Number of identified unique N-glycopeptides from mixture of AGP, fetuin and transferrin digests. LC-MS/MS measurements were carried out with various high CE choices (50%, 75%, 100%, 125%, 150%) combined with several different lowCE/highCE ratio (0.3, 0.5 and 0.7) and data analysis was performed using Byonic.

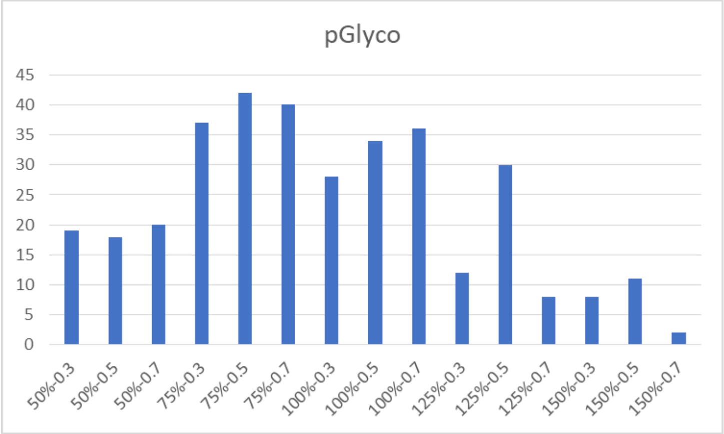

Fig. S2 Number of identified unique N-glycopeptides from mixture of AGP, fetuin and transferrin digests. LC-MS/MS measurements were carried out with various high CE choices (50%, 75%, 100%, 125% and 150%) combined with several different lowCE/highCE ratio (0.3, 0.5 and 0.7) and data analysis was performed using pGlyco3.0.

Time Fraction of High Energy Component

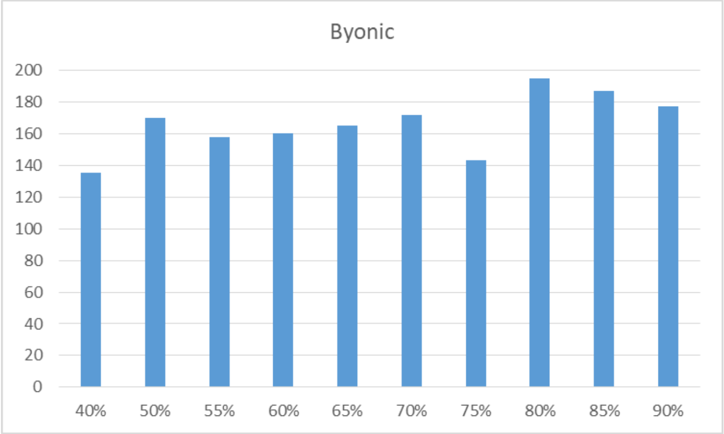

Fig. S3 Number of identified unique N-glycopeptides from mixture of AGP, fetuin and transferrin digests. LC-MS/MS measurements were carried out with various time fraction spent on the high CE condition and data analysis was performed using Byonic.

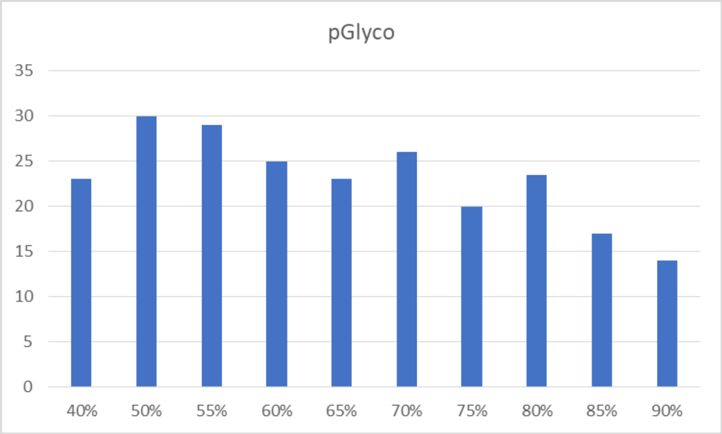

Fig. S4 Number of identified unique N-glycopeptides from mixture of AGP, fetuin and transferrin digests. LC-MS/MS measurements were carried out with various time fraction spent on the high CE condition and data analysis was performed using pGlyco3.0.

Higher Energy Component of the Optimal CE Setting for N-glycopeptides with ENGTISR and ENGTVSR Peptide Backbone Analyzed by pGlyco

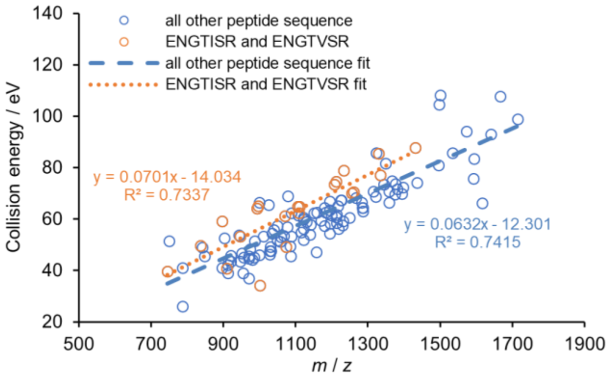

Fig. S5 Higher component of the optimal collision energies in eV as a function of m/z using pGlyco3.0 search engine. Orange circles indicate the optimum higher energy component for N-glycopeptides with ENGTISR or ENGTVSR peptide backbone, blue circles belong to the positions of all the other N-glycopeptide species. Dashed and dotted lines belong to the linear fits of the measured data points.

Example MS/MS Spectra Taken at CE Setting of Hinneburg et al. and Optimal CE Setting

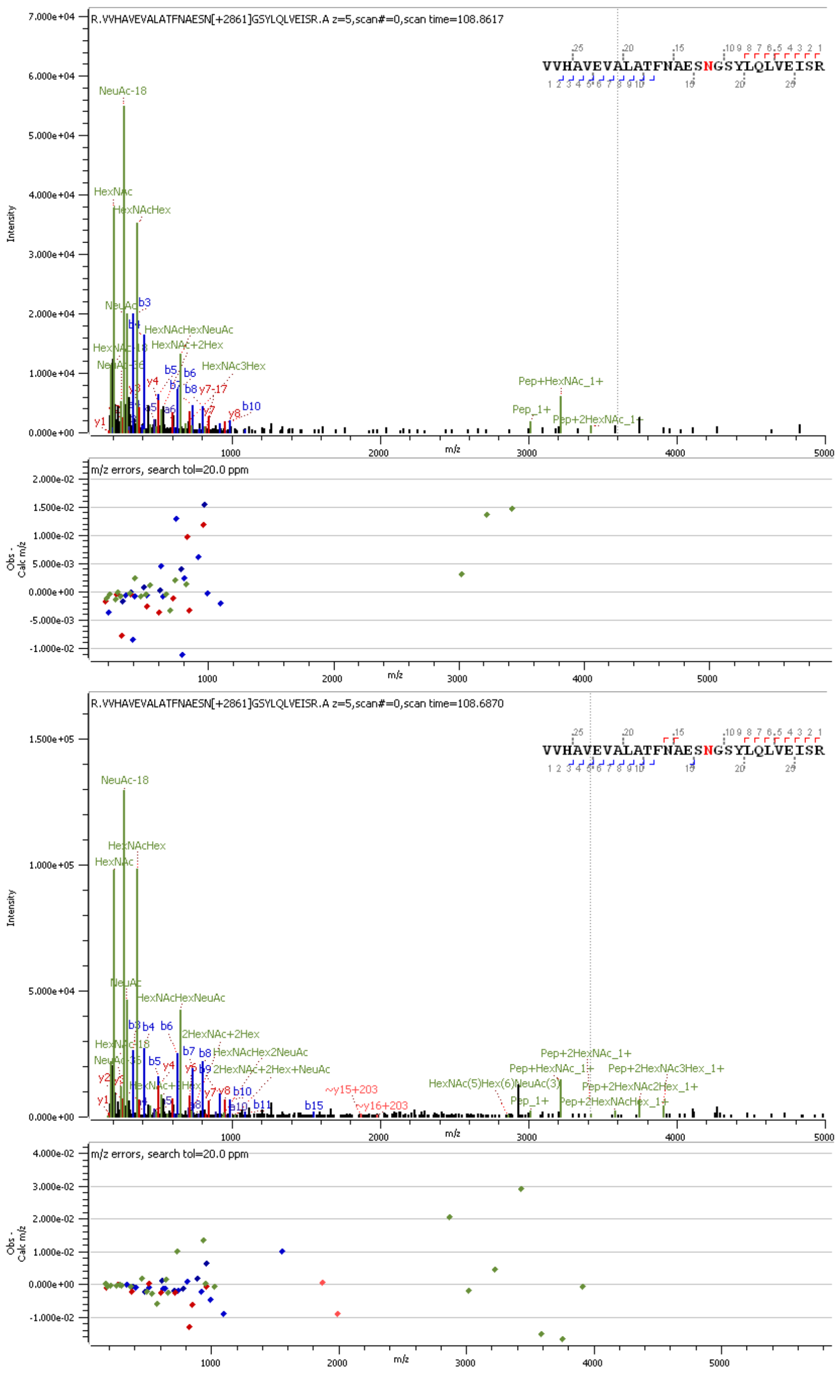

Fig. S6 Example MS/MS spectra of VVHAVEVALATFNAESNGSYLQLVEISR-HexNAc(5)Hex(6)NeuAc(3)$^{5+}$ N-glycopeptide (fetuin) at Hinneburg et al. setting (CE high component: 85 eV, Byonic score: 575, upper part) and our optimized setting (CE high component: 69.1 eV, Byonic score: 792, lower part).

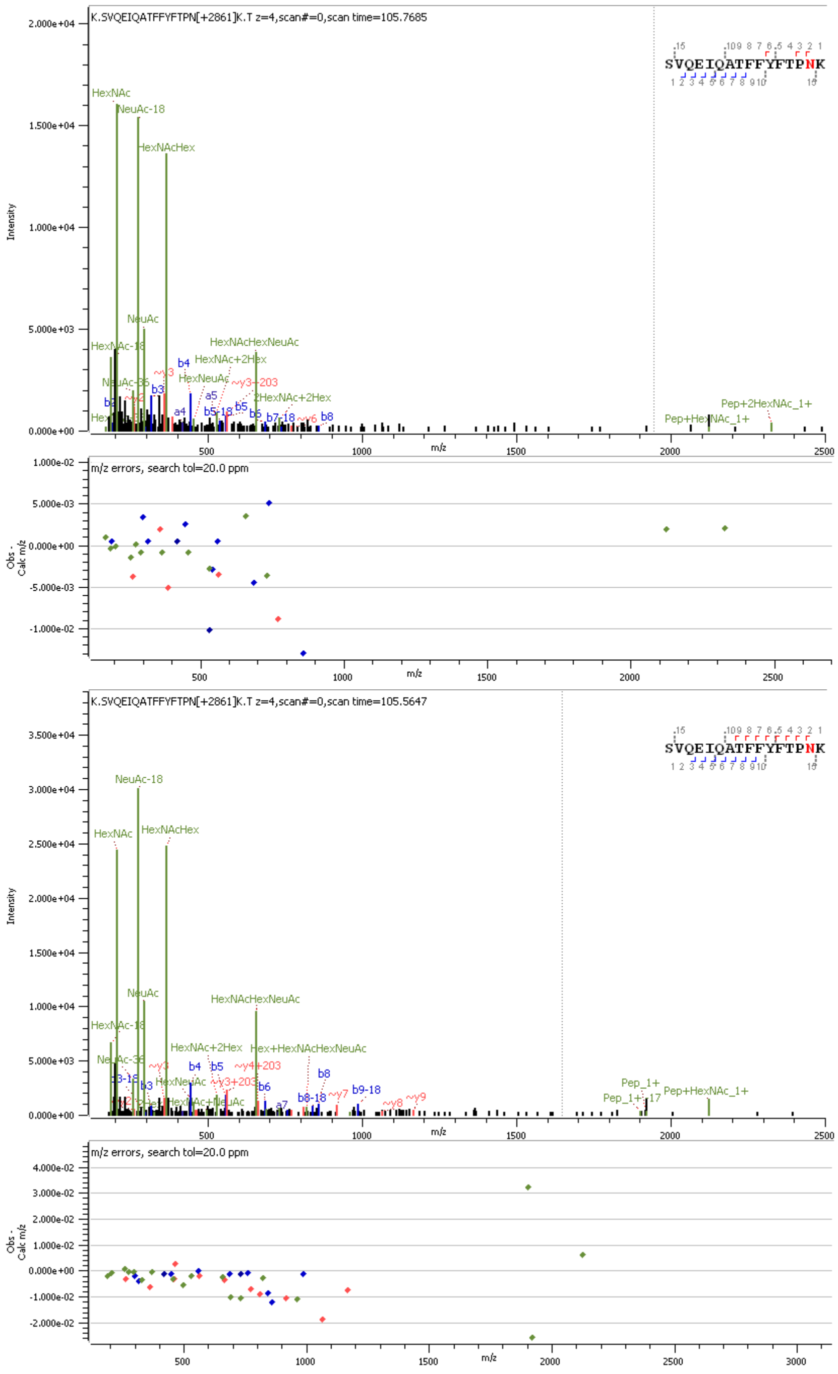

Fig. S7 Example MS/MS spectra of SVQEIQATFFYFTPNK-HexNAc(5)Hex(6)NeuAc(3)$^{4+}$ N-glycopeptide (AGP) at Hinneburg et al. setting (CE high component: 86.2 eV, Byonic score: 410, upper part) and our optimized setting (CE high component: 70.0 eV, Byonic score: 526, lower part).

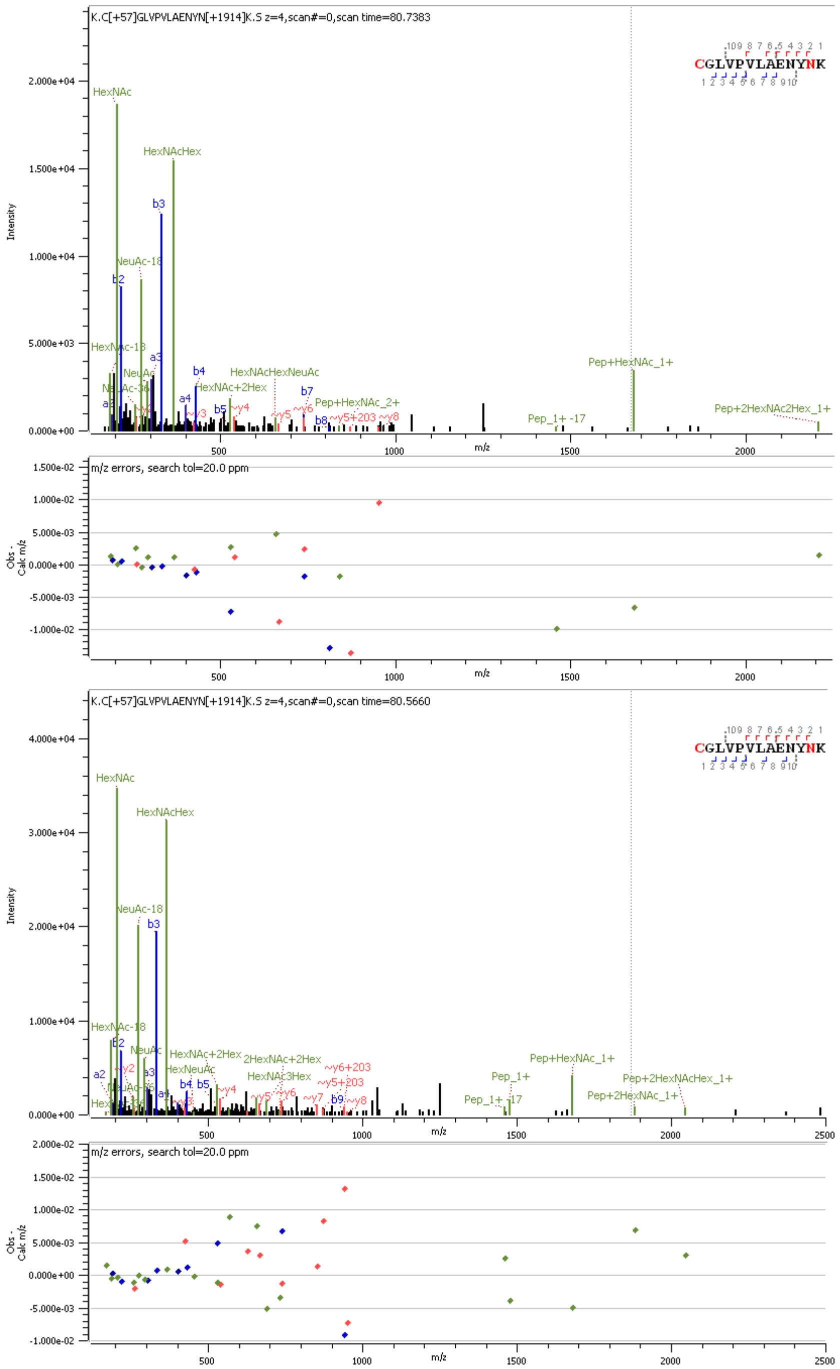

Fig. S8 Example MS/MS spectra of CGLVPVLAENYNK-HexNAc(4)Hex(5)NeuAc(1)$^{4+}$ N-glycopeptide (transferrin) at Hinneburg et al. setting (CE high component: 65.1 eV, Byonic score: 458, upper part) and our optimized setting (CE high component: 52.9 eV, Byonic score: 527, lower part).

Performance of Optimized Setting Analyzed by pGlyco

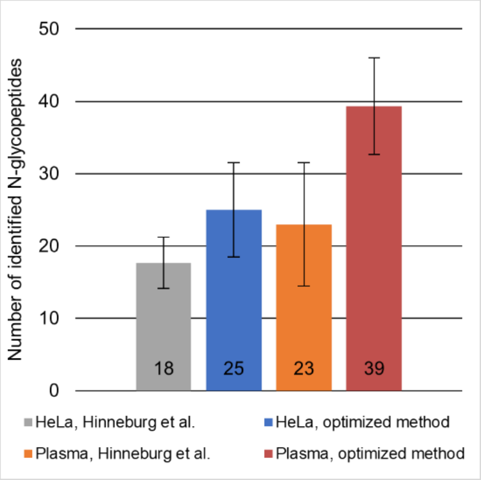

Fig. S9 Number of identified unique N-glycopeptides as average of three repeats analyzed by the pGlyco3.0 search engine. Error bars indicate ±1 standard deviation.

Results with a 3-step method

Table S2. Assessment of the potential impact of using 3 collision energy steps on the performance of glycopeptide analysis workflow. We used three different CE setups: Hinneburg et al.'s settings (referred to as "literature values" throughout), our optimized two-step method, and a three-step method consisting of our optimized low and high CEs with an additional 3rd step corresponding to their average, all three in equal time fractions. We determined the number of unique glycopeptide hits, and the average Byonic score on them on a sample consisting of a mixture of three glycoprotein digests (AGP, fetuin, transferrin).

	**# of identified unique N-glycopeptides**					**average Byonic score**				
	run					run				
**Method**	1st	2nd	3rd	4th	**Average**	1st	2nd	3rd	4th	**Average**
**Literature (Hinneburg et. al)**	307	216	264	265	**263.0**	388.4	351.8	356.4	348.5	**361.3**
**Optimized**	341	287	284	290	**300.5**	445.3	401.3	379.3	360.8	**396.7**
**Optimized with 3 steps**	177	299	279	285	**260.0**	309.0	370.1	343.2	355.5	**344.5**

Results on mAb Samples

Table S3. Comparison of the three experimental nano-LC-MS/MS methods tested on monoclonal antibody sample. Results are average of three repeats.

		Hinneburg et al.	optimized for glycoprotein standards	optimized for mAb
Byonic	# of identified unique N-glycopeptides	31	30	34
	average score	386	367	388
	average logProb	5.6	6.5	6.8
pGlyco	# of identified unique N-glycopeptides	19	28	25
	average score	15	21	18

List of Reference N-glycopeptides

Table S4. List of N-glycopeptides recommended for determination of optimal CE setting on other mass spectrometers.

Peptide Sequence	Glycan	Charge	Calculated m/z	Protein
QDQCIYNTTYLNVQR	HexNAc(6)Hex(7)NeuAc(1)	5	912.77	AGP
NEEYNK	HexNAc(5)Hex(6)NeuAc(3)	4	915.09	AGP
NEEYNK	HexNAc(5)Hex(6)Fuc(1)NeuAc(3)	4	951.61	AGP
WFYIASAFRNEEYNK	HexNAc(4)Hex(5)NeuAc(2)	4	1036.43	AGP
NEEYNK	HexNAc(4)Hex(5)Fuc(1)NeuAc(2)	3	1049.73	AGP
QNQCFYNSSYLNVQR	HexNAc(6)Hex(7)Fuc(1)NeuAc(3)	5	1059.42	AGP
QDQCIYNTTYLNVQR	HexNAc(6)Hex(7)	4	1067.94	AGP
QNQCFYNSSYLNVQR	HexNAc(6)Hex(7)NeuAc(4)	5	1088.42	AGP
QDQCIYNTTYLNVQR	HexNAc(6)Hex(7)Fuc(1)NeuAc(4)	5	1116.64	AGP
NEEYNK	HexNAc(5)Hex(6)NeuAc(2)	3	1122.76	AGP
CGLVPVLAENYNK	HexNAc(4)Hex(5)NeuAc(1)	3	1130.81	transferrin
NEEYNK	HexNAc(5)Hex(6)Fuc(1)NeuAc(2)	3	1171.44	AGP
KLCPDCPLLAPLNDSR	HexNAc(5)Hex(6)NeuAc(3)	4	1183.24	fetuin
QDQCIYNTTYLNVQR	HexNAc(7)Hex(8)Fuc(1)	4	1195.74	AGP
WFYIASAFRNEEYNK	HexNAc(5)Hex(6)NeuAc(3)	4	1200.48	AGP
NEEYNK	HexNAc(5)Hex(6)Fuc(1)NeuAc(3)	3	1268.47	AGP
SVQEIQATFFYFTPNKTEDTIFLR	HexNAc(6)Hex(7)NeuAc(4)	5	1283.34	AGP
QDQCIYNTTYLNVQR	HexNAc(6)Hex(7)Fuc(1)NeuAc(3)	4	1322.78	AGP
QQQHLFGSNVTDCSGNFCLFR	HexNAc(5)Hex(6)NeuAc(3)	4	1344.79	transferrin
QNQCFYNSSYLNVQR	HexNAc(6)Hex(7)NeuAc(4)	4	1360.28	AGP
QQQHLFGSNVTDCSGNFCLFR	HexNAc(5)Hex(6)Fuc(1)NeuAc(3)	4	1381.30	transferrin
LCPDCPLLAPLNDSR	HexNAc(5)Hex(6)NeuAc(3)	3	1534.62	fetuin
QQQHLFGSNVTDCSGNFCLFR	HexNAc(4)Hex(5)NeuAc(2)	3	1573.97	transferrin
